# Sensibility of the Skin

**Published:** 1887-12

**Authors:** 


### SENSIBILITY OF THE SKIN.

It is a matter of general surprise that would-be suicides are not disuaded from cutting their throats by the intense pain naturally considered inherent in the tissues.

In searching for the explanation of this fact, Prof. Brown-Sequard has proven that the slightest incision in the cervical region immediately destroys the sensibility of the anterior half of the neck, in all of its thickness; and also a sudden mechanical irritation of the skin of the neck and larnyx causes a paralysis of the great nerve centers, and consequent cessation of respiration and circulation.

In this way death by hanging, without suffocation, can be explained.—*Archives, Generales de Medicine.*

